# Improved Pullulan Production and Process Optimization Using Novel GA–ANN and GA–ANFIS Hybrid Statistical Tools

**DOI:** 10.3390/biom10010124

**Published:** 2020-01-10

**Authors:** Parul Badhwar, Ashwani Kumar, Ankush Yadav, Punit Kumar, Ritu Siwach, Deepak Chhabra, Kashyap Kumar Dubey

**Affiliations:** 1 Microbial Process Development Laboratory, University Institute of Engineering and Technology, Maharishi Dayanand University, Rohtak-124001, Haryana, India; b6parul@gmail.com (P.B.); punitdariyapur@gmail.com (P.K.); ritusiwach91@gmail.com (R.S.); 2 Optimization and Mechatronics Laboratory, Department of Mechanical Engineering, University Institute of Engineering and Technology, Maharishi Dayanand University, Rohtak-124001, Haryana, India; goswamiashwanikumar@gmail.com (A.K.);; 3 Bioprocess Engineering Laboratory, Department of Biotechnology, Central University of Haryana, Mahendergarh-123031, Haryana, India; ankushrao540@gmail.com

**Keywords:** Pullulan, genetic algorithm, artificial neural network, fermentation

## Abstract

Pullulan production from *Aureobasidium pullulans* was explored to increase yield. Non-linear hybrid mathematical tools for optimization of process variables as well as the pullulan yield were analyzed. The one variable at a time (OVAT) approach was used to optimize the maximum pullulan yield of 35.16 ± 0.29 g/L. The tools predicted maximum pullulan yields of 39.4918 g/L (genetic algorithm coupled with artificial neural network (GA–ANN)) and 36.0788 g/L (GA coupled with adaptive network based fuzzy inference system (GA–ANFIS)). The best regression value (0.94799) of the Levenberg–Marquardt (LM) algorithm for ANN and the epoch error (6.1055 × 10^−5^) for GA–ANFIS point towards prediction precision and potentiality of data training models. The process parameters provided by both the tools corresponding to their predicted yield were revalidated by experiments. Among the two of them GA–ANFIS results were replicated with 98.82% accuracy. Thus GA–ANFIS predicted an optimum pullulan yield of 36.0788 g/L with a substrate concentration of 49.94 g/L, incubation period of 182.39 h, temperature of 27.41 °C, pH of 6.99, and agitation speed of 190.08 rpm.

## 1. Introduction

Pullulan is a biopolymer of high commercial importance and utility [1]. Pullulan which is mostly available in powdered form, can also be formulated to thin films. These films have numerous applications in food, pharma, and healthcare industries. The most popular and commercially successful application of pullulan films is in Listerine^®^ mouth freshener. Pullulan has also received wider acceptance in the food sector, and due to its intensifying nature has become an accepted ingredient in soups, sauces, and beverages [2]. Pullulan can be used in pharmaceuticals, such as in coatings on pills and capsules, including sustained-release formulations [2].

For fermentative production of pullulan, *Aureobasidium pullulans* is the preferred microbial source. Pullulan as an exopolysaccharide is produced by *A. pullulans* in response to external pH change and nutrient deficiency [3]. *A. pullulans*, as well as its upstream processing, has been the subject of an increasing body of research in fermentation studies. For an efficacious full fermenter system analysis, research must cover certain fermentation variables, such as aeration rate, shear rate, and medium composition, whereas for a primary bench-top evaluation study, key steps include optimizing medium content and their concentration along with culture conditions. Many studies report optimization of pullulan production by altering the nutrient content of the medium [4]; fermentation conditions such as temperature [5]; minerals such as “Zn^2+^, Fe^2+^, Mn^2+^, Ca^2+^, and Cu^2+^ [6]; pH [7], and process technology [8]. Carbon and nitrogen, being the two most important medium nutrients, have also been studies extensively. In these studies, the conventional carbon and nitrogen source for the medium were glucose and peptone, respectively. The medium configuration with a specific *A. pullulans* variant produces 20–50.2 g/L of pullulan [9,10]. An entire body of literature is dedicated to the concept of alternate carbon/nitrogen sources. These sources are said to be cheap and wholesome sources of carbon/nitrogen [11,12], and several reports include the use of agro-industrial waste. To enhance the yield of pullulan (up to 85 g/L) and design an economic process, de-oiled jatropha seed cake has been exploited as the nitrogen source along with glucose (carbon source) [13]. Jaggery, as a cheap and alternate source of glucose, along with de-oiled jatropha seed cake [14], enhanced the yield up to 65 g/L.

A typical approach in medium optimization involves the change of one process variable at a time and the study of its effect on the whole process, leaving other variables as unspecified constants. This approach describes individual effects of the variables, whereas a bio-process is a cumulative effect of multiple process variables affecting the final result simultaneously. Thus, the classical approach fails to produce significant results and also consumes more time. The approach becomes even more expensive, as well as extensive, when multiple or large numbers of variables are under scrutiny simultaneously. Unconventional methods using mathematical algorithms are the answer to the weaknesses of conventional optimization approaches. Algorithms are reliable, reproducible, and quick to produce validated results. Such modeling processes are used to study the cumulative effects of all the factors influencing a process by simultaneously varying them, all within a limited number of experimental runs. These efficient methods have found a strong foothold in current optimization and modeling studies. In a 2012 study, an artificial neural network coupled with a genetic algorithm (GA–ANN) was used to positively optimize seven process parameters for citric acid production from *Aspergillus niger* MCBN297 [15]. An ANFIS model with five neurons and one input layer, seven neurons with one hidden layer, and one output with one neuron accurately predicted polygalacturonase (PG) production by *Bacillus subtilis* [16].

**Figure 1:**
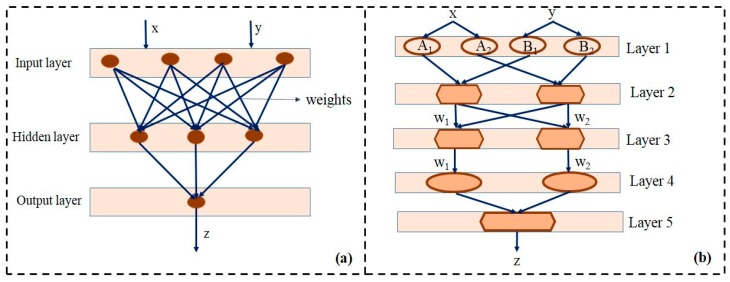
Schematics of artificial neural network (ANN) (**a**) and adaptive network based fuzzy inference system (ANFIS) (**b**) architecture. “x” and “y” represent the input variable whereas the “z” characterizes the output variable in both the ANN and ANFIS architecture. The oval structures in layer 1 and layer 4 represent the adaptive nodes. The pentagonal structures in layer 2, 3, and 5 are the predefined fixed nodes.

Artificial neural network (ANN) algorithms have achieved a dependable position in the modeling of multifaceted biological systems. In contrast to conventional statistical techniques, ANN performs exceptionally well in pattern recognition and modeling of random relationships of mass variables [17]. The basic framework of ANN is quite similar to the human brain (Figure 1a). It includes organized layers of neurons, namely the input layer neuron (symbolizes input data), the output layer neuron (response of the input data), and the hidden layer neuron (for processing of input data). These neurons are connected by scalar functions known as weights. The learning process of an ANN algorithm is performed by revising weights through continuous iterations and error minimization [18]. Another artificial intelligence technique that has been gaining importance in the field of bioprocess optimization is the genetic algorithm (GA). GAs imitates the Darwin evolutionary theory, “Survival of the fittest”, while probing an objective function. GAs split the input variable by using discrete string codes; these functions further augment the group’s fittest function. Genetic algorithms can be used to improve the input array of the neural network models to find an optimum arrangement for output prediction. In a GA–ANN optimization operation, the ANN model algorithm aids in computing the fitness function of an input string followed by reproduction, mutation, and crossover operations in multiple directions towards optimization. The algorithm is aborted only when the conjunction criterion is fulfilled. These two (ANN and GA) very imperative algorithms when integrated together generate a potent tool for modeling and process optimization studies [19].

Jang [20] proposed a highly significant non-linear neuro-fuzzy model called adaptive network based fuzzy inference system (ANFIS). Takagi and Sugeno’s fuzzy rule “if–then” form the base rule of ANFIS followed by linear functions of input. This method reduces the number of obligatory fuzzy rules. Thus, the approach takes advantage of both the fuzzy logic and neural networks, i.e., the algorithm incorporates elusive data by fuzzy logic and concurrent learning through neural networks (Figure 1).

Assume that the fuzzy inference system has two inputs, x and y, and one output, z (Figure 1b). The first-order Sugeno fuzzy model has the following rules:

Rule 1:

If x is A1 and y is B1, then f1 = p1x + q1y + r1

Rule 2:

If x is A2 and y is B2, then f2 = p2x + q2y + r2

Considering a simple fuzzy interference system of two inputs, x and y, and one output, z. A and B are the linguistic labels associated with the respective node function (1 and 2). Then p, q, and r are the consequent parameter sets assigned in the ANFIS layers [20]. The building blocks of a FIS form the layered ANFIS architecture (Figure 1b) as follows:

Layer 1: The fuzzification layer denotes the membership functions (MFs) to every input.

Layer 2: The rule layer executes the fuzzy AND precursors parts of the fuzzy rules.

Layer 3: Normalizes the MFs. 

Layer 4: Denotes the decision-making unit.

Layer 5: Denotes a de-fuzzification interface.

Both of these artificial learning tools, namely ANN and ANFIS, have been successfully applied to biological systems as well as optimization of bioprocesses [21]. Several studies have reported process optimization by either ANN or ANFIS tools for the production of enzymes, explicitly protease [22], laccase [23], polygalactonase [24], arginine deaminase [25], and hydantoinase [19]. A limited number of studies has explored the feasibility of hybrid, non-linear modeling techniques with fermentation processes. In this report, both the genetic algorithms–artificial neural network (GA–ANN) and genetic algorithms–adaptive network based fuzzy inference system (GA–ANFIS) were applied individually to optimize the process parameters as well as pullulan production by *A. pullulans.* The models were analyzed on the basis of reproducibility and synchrony with experimental performance.

## 2. Materials and Methods

### 2.1. Growth and Culture of Aureobasidium Pullulans

For growth as well as fermentation of *A. pullulans,* only analytical grade reagents were used in this study. All the culture medium components were procured from Hi-Media (December 2018, Mumbai, India) and Sigma Aldrich (Bangalore, India). *A. pullulans* was procured from Microbial Type Culture Collection (MTCC 1991, Chandigarh, India). The microorganism was revived, grown, and maintained in YPD medium (yeast extract 10 g/L, peptone 20 g/L, dextrose 20 g/L, and agar 15 g/L at pH 7.0) and incubated at 28 °C [26]. The microorganism was sub-cultured every 3 weeks. By revivifying and sifting, microbial colonies with less pigmentation or no melanin formed were picked and sub-cultured to perform the following experiments: The inoculum was prepared by growing *A. pullulan* in growth medium containing 50 g/L sucrose, 2 g/L di-potassium phosphate, 1 g/L ammonium sulfate, 0.05 g/L magnesium sulfate heptahydrate, 0.5 g/L sodium chloride, 0.01 g/L ferrous sulfate, 0.01 g/L manganese (II) sulfate, and 0.01 g/L zinc sulfate at pH 7.0 ± 0.1 in an incubator shaker at 28 °C and 200 rpm for 48 h [23]. The fermentation medium with 50 g/L sucrose; 2 g/L yeast extract; 1.5 g/L NaCl; 5 g/L K_2_HPO_4_; and 0.2 g/L MgSO_4_.7H_2_O was inoculated by the seed culture (10% *v*/*v*). The fermentation was carried out in a shake flask at 200 rpm and 28 °C for 168 h [23].

### 2.2. Optimization of Fermentation Variables

For each and every fermentation process, temperature (°C), pH, duration of fermentation (h), substrate concentration (g/L), and agitation speed (rpm) are considered as vital parameters. In the present study also, the cumulative effect of all the 5 variables was studied on pullulan production using *A. pullulans* in broth medium. The optimization study was carried out in 250 mL conical flasks with a 100 mL working volume.

#### 2.2.1. Process Optimization by Conformist Approach

The conventional one process variable at a time (OVAT) approach was adopted for the optimization of the fermentation process variables (temperature, pH, duration of fermentation, substrate concentration, and agitation speed). Sucrose was selected as the primary substrate (carbon source) for pullulan production. The concentration of sucrose plays a vital role in the morphology and survival of *A. pullulans* under laboratory conditions, which directly correlates to the pullulan yield. Thus, an inclusive stretch of sucrose concentration (25–100 g/L) was considered for the optimization study. Other process parameters, such as pH (5–8), incubation temperature (25–30 °C), duration of fermentation (120–240 h), and agitation speed (50–250) were individually studied by fluctuating one factor at a time. To determine the effects of process variables on pullulan yield, the fermentation was carried out in 250 mL conical flasks with a 100 mL working volume. For more precision and accuracy, all sets of experiment were performed in triplicate.

#### 2.2.2. Development of Artificial Neural Networking (ANN) Model for Optimization of Process Variables

The simulation work was performed in MATLAB 7.11.0 (R2010b, The MathWorks, Inc., Natick, Massachusetts, MA, USA, Trix LABoratory). For the toolbox, a fitting problem of the input data was loaded, which was a 5 × 50 matrix (.mat file from the workspace), representing the following static data: 50 samples of 5 elements, namely substrate (g/L), time (h), temperature (°C), pH, and agitation speed (rotation per minute (rpm)). Then the target input data were loaded describing the preferred network output, which was a 1 × 50 matrix (.mat file from the workspace), representing static data of 50 samples of 1 element that is the weight of pullulan (g/L). Post data selection, the crucial steps of validation and data assessment were performed by random division of 17 selected samples using a standard approach, such as 34 samples for training, 8 for validation, and 8 for testing. Frequent training and testing of selected samples were performed with simultaneous network improvisation learning from the errors. The cycle ceased to perform or was terminated from further training only when the hypothesis ceased to move forward. In order to define a fitting neural network in the present study, 10 hidden layers were stipulated (an ANN defined default parameter). The system offers different training algorithms, such as scaled conjugate gradient back propagation (trainscg), Levenberg–Marquardt back propagation (trainlm), and resilient back propagation (trainrp). The Levenberg–Marquardt back propagation (trainlm) algorithm was the preferred choice to train the network and for the fitting of inputs and target. The regression measure (R value) describes the relationship between inputs and the target. A value near to 0 represents a random relationship, whereas a close relationship is represented by values near to 1.

#### 2.2.3. Implementing the GA–ANN Model to Optimize the Process Parameters for Pullulan Yield

A hybrid GA–ANN approach was employed for the maximization of pullulan weight and providing the optimized process parameters, within their loaded upper and lower bound limits in GA. The net file (.mat format) obtained from the perfect fitted ANN model was loaded for the desired fitness function. The fitness function was loaded in the form of @fitnessfun and the file fitnessfun.m calculated the fitness function. For further processing of the 5 variables, linear equalities, bounds and integer variable indices, and other constraints were defined. Along with those constraints, population size of 100, stopping criteria of 20 generations, mutation rate of 0.05, crossover fraction of 0.9, elite count of 1, constraint dependent type creation function, and other default parameters were also defined. The two best suited models of the ANN algorithm designated as ‘trainlm 1 and 2′ were subjected to a parallel exercise to accomplish optimized results. The results were obtained in the form of best and mean values of pullulan weight and corresponding input process parameters.

#### 2.2.4. Development of ANFIS Model for Experimental Data Training

A neuro-fuzzy designer app was loaded with the .mat file. The file contained a 50 × 6 matrix of static data representing 50 samples of 5 input elements, namely substrate (g/L), time (h), temperature (°C), pH, and agitation speed (rpm), and one output element, which was pullulan yield (g/L). The grid-partition method was chosen for generating the FIS of the Sugeno type. For data training, three constant type membership functions (MFs) were chosen for each input variable and other default parameters, such as Hybrid train FIS optimization method, 0 error tolerance, 243 rules, and 3 numbers of iterations. For further data assessment, 5 different MFs, namely gaussmf, trimf, gbellmf, gauss2mf, and dsigmf, were picked. These 5 MFs were performed in succession to fabricate a reformed FIS for data training in five assorted ways. The different FIS files (.fis), corresponding to five individual MFs, were saved for further exclusive integration into a fitness function. The fitness functions were later used by GA to optimize the process parameters.

#### 2.2.5. Optimization of Process Parameters for Pullulan Production Using the GA–ANFIS Model

Similar to the GA–ANN model, to optimize the input parameters for the enhancement of pullulan yield, the established ANFIS model (. fis File) was combined as a fitness function in the GA. As per the preset GA problem, keeping all the default parameters the same as earlier, different ANFIS models were trained for five individual MFs in order to optimize the process parameters and target using a hybrid GA–ANFIS approach.

### 2.3. Extraction and Estimation of Pullulan Yield

For biomass separation and pullulan extraction from the fermented broth, arrays of unit operations were performed. For preliminary separation of biomass, centrifugation was performed at 10,000 rpm for 20 min (RCF: 5070) at room temperature. The cell pellet was suspended into 10 mL distilled water and again centrifuged at 10,000 rpm for 20 min; the whole process was performed thrice. The pellet was autoclaved and discarded, and all the supernatants obtained via washing and centrifugation were pooled for further downstream processing. Organic solvent precipitation was performed to the cooled supernatant (4 °C for 3 h) to extract the dissolved pullulan. Ethanol was used as the organic solvent for the downstream processing. A double volume of cold ethanol was added to cool the supernatant in a stepwise manner. Constant mixing was performed by magnetic stirrer. Through the process, pullulan precipitated at the bottom of the flask. The flask was left to stand still at 4 °C for the precipitate to suitably accumulate at the bottom [26]. The precipitate was dried on pre-weighed filter paper at 50 °C. The dried pullulan weight (g/L) determined the pullulan yield [27].

## 3. Results and Discussion

### 3.1. Optimization of Various Production Parameters Using the OVAT Approach

#### 3.1.1. Substrate Optimization by the OVAT Approach

Being ubiquitous, the yeast-like fungus, *A. pullulans*, can survive on a variety of substrates. It has also been claimed by many researchers that carbon source might be a significant factor in melanin pigmentation found in many strains of the microbe [28]. Sucrose, fructose, maltose, xylose, and glucose were the most common time and tested carbon sources for fermentative production of pullulan [29]. Through extensive study of the metabolic pathway of sucrose in pullulan fermentation, it was found that sucrose did not cleave into glucose and fructose in the medium [30,31]. In the present study also, sucrose was adopted as the sole substrate or carbon source for pullulan production. For optimization of sucrose level in this medium, the conventional one variable at a time (OVAT) approach was adopted. An inclusive stretch of sucrose concentrations (25–100 g/L; 25, 30, 35, 40, 45, 50, 55, 60, 65, 70, 75, 80, 85, 90, 95, 100) was considered for the optimization study. Meanwhile other important variables including temperature, pH, duration of fermentation, and agitation speed were kept constant. Initially, pullulan production increased with increases in the sucrose concentration (25–40 g/L), but with further increases in sucrose concentration, the pullulan yield tended to cease; 45.6 ± 0.14 g/L of sucrose produced a maximum of 34.89 ± 0.17 g/L of pullulan. The results obtained were in perfect relation with the literature; Shin et al. [32] initially found the inhibitory effect of high sucrose concentration. It was further assessed that this phenomenon could be due to low water activity and osmotic effects [33].

#### 3.1.2. Optimization of Temperature by the OVAT Approach

Temperature is an integral factor of every fermentation process having to do with the fermentation of *A. pullulans.* Being a ubiquitous fungus, *A. pullulans* can survive in a plethora of temperature conditions; the effect of temperature upon morphology and consequently on pullulan production was described by McNeil and Kristiansen [34]. They found that 24 °C is the optimum temperature for maximum pullulan production. The variation in yield of exo-polysaccharide (EPS) with temperature may be due to morphology as well as temperature [34]. The temperature range under observation for *A. pullulans* (MTCC 1991) was also chosen around the ambient temperature range 25–30 °C (25, 26, 27, 28, 29, and 30 °C). The maximum production of pullulan (35.47 ± 0.25 g/L) was observed at 27.5 °C under controlled laboratory conditions, optimized by the OVAT approach.

#### 3.1.3. Optimization of pH by the OVAT Approach

It was observed that the pH of the fermentation system may not have a direct effect on pullulan yield or EPS secretion into the medium, but it does affect the growth of *A. pullulans.* Shingel and Kirill [7] offered a broad range (5.5–7.5) of optimal pH for pullulan production. It was further observed that optimum pH for the cultivation of *A. pullulans* differs from the pH required for maximum pullulan production. Taking direction from the literature, the experiments were designed in the range of pH 5–8 (5, 5.5, 6, 6.5, 7, 7.5, 8, and 8.5). With the initial pH range, lesser microbial colonies were observed, leading to a low yield of pullulan. The maximum population of *A. pullulans* was observed between the pH ranges 6.5–7.5. Analogous to microbial growth, maximum pullulan yield was also observed with pH 6.5–7.5 (34.86 ± 0.34 g/L).

#### 3.1.4. Optimization of Duration of Fermentation (Time) by the OVAT Approach

Incubation period or time duration of a fermentation process describes the time required by the microorganism for its maximum growth and metabolite production by consuming the medium content. Multiple studies have offered optimization of fermentation medium by various statistical methods [35]. To assess the effect of incubation time over a span of 120–240 h, 11 variable time halts were selected, i.e., 120, 132, 144, 156, 168, 180, 192, 204, 216, 228, and 240. At each and every standstill, the microbial growth was observed followed by the pullulan yield evaluation. The microbial growth was measured in terms of dry cell weight (DCW), and a maximum of 219.90 mg/L of DCW was observed in the time range 144–200 h with minor oscillations. This time range was further analyzed for pullulan yield; after 180 h of incubation, a maximum pullulan yield of 35.43 ± 0.42 g/L was observed.

#### 3.1.5. Optimization of Agitation Speed by the OVAT Approach

Agitation speed has a direct effect on the shape and biomass distribution in the medium. Agitation at 50, 100, 150, and 200 rotations per minute (rpm) was performed to analyze its effect on the fermentation of *A. pullulans* and pullulan yield. Microbial colonies of *A. pullulans* have shown visible effects of agitation rate in terms of the size, shape, and distribution in the broth medium. Another distinguished effect of agitation was observed with the formation of a matt-like structure on the periphery of the medium sticking to the glass wall. The transformation of yeast to mycelial growth forms was clearly visible with this. With increased agitation speed, colonies tended to reduce in size but increase their overall colony count. These single cell colonies of *A. pullulans* changed their globular shape to diffused hyphae and slender colonies but with more colonies in number with higher agitation speed (200 rpm). Thus, at higher agitation speed, higher numbers of colonies were observed producing more amounts of pullulan (33.83 ± 0.17 g/L).

### 3.2. Optimization of Fermentation Process Variable and Pullulan Yield by Hybrid Learning Tools

By the conventional one variable at a time approach, the maximum pullulan yield observed was 35.16 ± 0.29 g/L. The yield was noteworthily improved compared to yields documented in the present literature on pullulan production, but the approach was significantly futile in terms of reciprocity between process variables. Nor were the effects of plausible cumulative effects of these variables considered, and if taken into account, it will be very difficult to conceptualize and implement all these sets of experiments manually. Artificial learning tools come in handy in such complicated situations. GA–ANN and GA–ANFIS were chosen in the present study to optimize the input bioprocess variables and amplify the output function (pullulan yield).

#### 3.2.1. ANN Model for Training of Experimental Data

The simulation work was done in MATLAB 7.11.0 (R2010b). The command “nnstart” was employed for the open neural network wizard followed by the selection of a fitting app for input–output and curve fitting. Fitting functions were deliberately executed by neural networks with their pronounced fitting capacities. Neural networks are capable of fitting any realistic specific function. The fitting problem presented to the toolbox for the present study was a 5 × 50 matrix (.mat file from the workspace), representing the following static data: 50 samples of five elements, namely substrate (g/L), time (h), temperature (°C), pH, and agitation speed (rpm). Then target data was represented by a 1 × 50 matrix (.mat file from the workspace), representing static data, namely 50 samples of one element, i.e., pullulan weight (g/L). Among the available training algorithms Levenberg–Marquardt back-propagation (trainlm) was adopted for further training and data fitting operations. As the preliminary set of conditions defines the specific outcome results, an explicit set of conditions producing the best results were further analyzed for different plots, for instance plot error histogram, plot fit, and plot regression.

**Figure 2:**
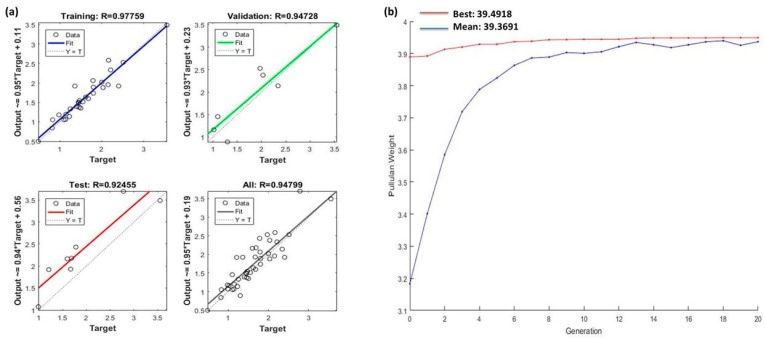
Regression plot obtained by training and optimization of the genetic algorithm (GA)–ANN tool. The plots represent the correlation between outputs and targets corresponding to the optimal Levenberg–Marquardt algorithm (**a**). Convergence of GA–ANN for maximizing pullulan weight (**b**).

In order to evaluate the relationship between outputs and targets, the R values (regression measures) were analyzed. The R values also define the prediction precision by an algorithm. An R value near to 0 characterized a random relationship, whereas for a close relationship, R values were found to be close to 1. The regression plots illustrate the correlation between input and target (Figure 2a) corresponding to an optimal Levenberg–Marquardt algorithm. The R values for training, validation, and testing were 0.977, 0.947, and 0.924 respectively, indicating a good relation between test and validation results. Among the two Levenberg–Marquardt back-propagation training algorithms (trainlm1 and trainlm2), the best results for different regression values like training R, validation on R, test R, and all R are enlisted in Table 1. Finally, the generated regression diagrams and results were saved in the form of inputs, outputs, error, network (net), performance, and data set information (info) to MATLAB. In order to optimize reaction variables, the results attained from the artificial neural network (ANN) were further used in the genetic algorithm (GA).

#### 3.2.2. GA–ANN Model Executed to Optimize the Process Parameters for Pullulan Weight

To devise a maximum pullulan yield function, a hybrid GA–ANN approach was designed. The upper and lower constraints of process input variables were loaded in GA. The net file (.mat format) obtained from the ANN model was integrated with the fitness function and acted as the fitness function for GA. The ideal plots were obtained by a rank scaling function for fitness scaling, double vector type population to population size 100, stopping criteria of 20 generations, mutation rate as 0.05, crossover fraction as 0.9, elite count as 1, constraint dependent type creation function, and other default parameters. The same fitness functions were applied to two different ANN algorithms (trainlm1 and trainlm2) to be optimized. The results were obtained in the form of best and mean values of pullulan yield (Table 2).

The conclusion can be drawn from the assessment of GA–ANN results that Levenberg–Marquardt (trainlm) was the best suited algorithm for data training, and by integration with GA it provided the maximum value of pullulan weight (39.4918 g/L, as shown in Figure 2b), corresponding to various optimum process parameters as substrate: 48.9 g/L, incubation time 172.62 h, temperature 26.89 °C, pH 6.56, and agitation speed 224.85 rpm.

#### 3.2.3. ANFIS Model for the Experimental Data Training

As opposed to ANN, the ANFIS (adaptive network-based fuzzy inference system) model was developed and employed to find an ideal scheme that best fit the experimental data in the fuzzy system. Alzoubi et al. [36] also performed a similar study; they compared various integrating algorithm models like ICA-ANN, GA–ANN, PSO-ANN, and ANFIS and found that the GA–ANN method was the best among them for energy consumption predictions for irrigation land leveling, due to its low RSME and high R^2^ values. A concrete accomplishment of GA–ANN and GA–ANFIS tools for optimization of process parameters for xylanase bio-bleaching of mixed hardwood pulp was observed with a 28.05% increment in reducing sugar content in compared to un-optimized conditions of 21.99 mg/g [37].

**Figure 3:**
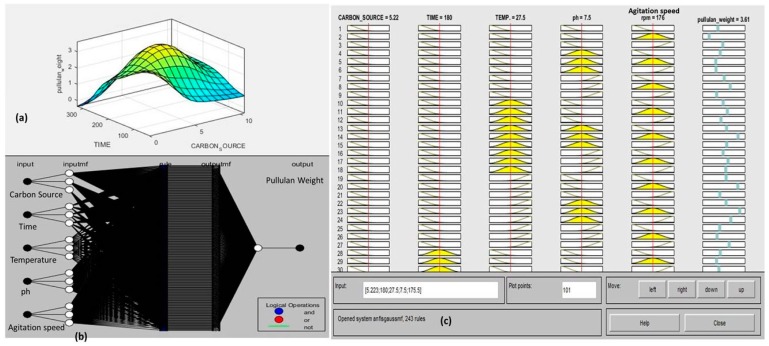
Training and optimization plots by the ANFIS tool for the optimization of pullulan yield. (**a**) ANFIS response surface figure. (**b**) ANFIS model structure for ‘trimf’. (**c**) Training routine of process variable (input) by ANFIS training rules.

ANFIS combines the best of the two artificial learning tools, i.e., neural networks and fuzzy systems. The tool is considered more potent with enhanced smoothness as well as interpolation flexibility. For input data (50 × 6 matrix, representing static data: 50 samples of five input elements, namely carbon source (g/L), time (h), temperature (°C), pH, agitation speed (rpm) and one output element that is pullulan weight (gm/100 mL)) training a constant type membership function (MF) for each input variable and other default parameters like hybrid train FIS optimization method, 0 error tolerance, 243 rules, and 3 as the number of iterations were chosen. It was not possible to attain an absolute data fitting with concurrent data training; hence, for every training trial a non-zero value of training error occurred (as shown in Figure 3a). The deviation of output was defined by the variation in any of the two selected inputs; the correlation was specified by an ANFIS response surface plot. Here the topmost point on the response surface plot elucidated the maximization of the target value (pullulan yield) (Figure 3a). Only after acquisition of tractable results, performance of FIS was tested against training. The model structure represented the interlinking among different inputs: input MFs, rules, output MFs, and outputs (Figure 3b). The rule viewer of the fuzzy inference system was utilized to understand the entire outgrowth of the process from commencement until the process was concluded (shown in Figure 3c).

#### 3.2.4. GA–ANFIS Model for Optimization of Process Parameters and Pullulan Weight

**Figure 4:**
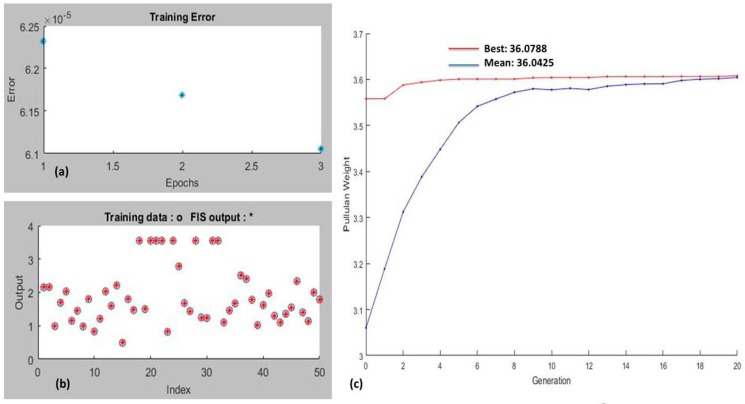
(**a**) Training error at three different epochs. (**b**) Training performance of FIS. (**c**) Convergence of GA–ANFIS for maximizing pullulan weight.

As per the predefined GA problem, all the default parameters were kept similar to the former. While training the ANFIS model, to enhance the prediction accuracy and subsequently decrease the prediction error, numerous plotting of the input dataset took place. The minimum obligatory number of repetitions to perform the mapping function was termed the epoch. As seen in Figure 4a, it was inferred that three epochs were essential to achieve the training process. For the learning capability of FIS, a function was defined by the overlapping position of a red star over the white circles; here, the prominence of overlapping positions also signified better fitting of data training trials (Figure 4b). Five different MFs, namely Gaussian curve membership function (gaussmf), triangular-shaped membership function (trimf), generalized bell-shaped membership function (gbellmf), Gaussian combination membership function (gauss2mf), and difference between two sigmoidal membership functions (dsigmf) were trained to optimize process parameters and targets pullulan yield. Various ANFIS training MFs were chosen, and the resultant optimized GA process parameters and yields are enlisted in Table 3. From the optimized results obtained through the GA–ANFIS approach, it can be concluded that among the various chosen MFs, the Gaussian membership function (gaussmf) was most appropriate for data training as it maximized the value of pullulan weight up to 36.0788 g/L with mean pullulan weight of 36.042 g/L (Table 3; Figure 4c). The other process parameters optimized by the gaussmf MF hybrid GA–ANFIS tool were substrate consumption 49.94 (g/L) at temperature 27.41 °C, with agitation speed 190.08 rpm at pH 6.99 and incubation period 182.39 h (Table 3).

After comparing the results obtained for optimization of pullulan weight using two different training approaches, namely ANN and ANFIS followed by GA, it can also be concluded that training of experimental data through the ANFIS model using gaussmf MF provided better results as compared to the ANN model, which were in near proximity to the laboratory experimental results.

#### 3.2.5. Comparative Analysis of the Artificial Intelligence and Simulation Approaches Employed (GA–ANN and GA–ANFIS)

The prediction accuracy of the two hybrid non-linear learning tools (GA–ANN and GA–ANFIS) are juxtaposed in Table 4. During several experimental runs, the pullulan yield achieved an optimum value of 35.5 g/L, corresponding to values of various input parameters such as carbon source (45.00 g/L), incubation time (180 h), temperature (27.5 °C), pH (7.5), and agitation speed (200 rpm). Using the ANN based data training and GA based optimization, the hybrid GA–ANN tool centered on the Levenberg–Marquardt training model and produced the improved value of pullulan weight of 39.4918 g/L. The tool optimized the values of analogous input parameters such as carbon source (48.9 g/L), incubation time (172.62 h), temperature (26.89 °C), pH (6.56), and agitation speed (224.85 rpm). The optimized input parameters provided by the GA–ANN approach was again tested by the experimental fermentation runs and thus provided the value of pullulan weight in the range of 37.642 ± 0.521 g/L with an accuracy of 95.32%. In order to achieve more concrete and optimized results of pullulan yield, the GA–ANFIS approach was also used. Training of experimental fermentation was done through the ANFIS model using gaussmf MF and optimized by the GA function tool. GA–ANFIS optimized the value of pullulan weight to 36.0788 g/L, corresponding to the values of various input parameters such as carbon source (49.94 g/L), time (182.39 h), temperature (27.41 °C), pH (6.99), and agitation speed (190.08 rpm). Again, the optimized input parameters provided by the GA–ANFIS approach were tested by the laboratory experimental runs and thus provided the value of pullulan weight in the range of 35.652 ± 0.348 g/L with an accuracy of 98.82%. The optimization tool, GA–ANFIS, provided more concrete results than GA–ANN and in closer approximation to experimental values with a higher percentage of accuracy.

## 4. Conclusions

Pullulan is a commercially valuable biopolymer in high demand. Due to its forthcoming supplementary requirement, the fermentative production of pullulan by *A. pullulans* has been optimized by many statistical tools including RSM, Plackett–Burman, Box–Behnken design, etc.

A non-linear, dynamic model for process variables as well as pullulan yield was successfully devised by artificial intelligence and simulation approaches (GA–ANN and GA–ANFIS). The enhanced productivity (12.34% increment in pullulan yield) and precise process variables provide by the hybrid tools increased the efficacy of the whole process.

The optimized pullulan yield provided by GA–ANN (39.4918 g/L) and GA–ANFIS (36.0788 g/L) was in perfect synchrony with experimental data as well as the literature. The prediction accuracy of GA–ANN was observed to be 95.32%, whereas by GA–ANFIS it was 98.82%. The optimization tool, GA–ANFIS, provided more concrete results than did GA–ANN and in closer approximation to experimental values with a higher percentage of accuracy.

The simple yet consistent medium composition and fermentation conditions provided by the optimization tools made the whole bioprocess more sophisticated, reliable, reproducible, and economic.

## Figures and Tables

**Table 1:** Significant regression ‘R’ values corresponding to Levenberg–Marquardt training algorithms ‘trainlm 1 and 2′.

Sr. No	Training Algorithm	Code Name Used	Training: R	Validation: R	Test: R	All: R
1	Levenberg–Marquardt (LM)	trainlm1	0.97759	0.94728	0.92455	0.94799
2	Levenberg–Marquardt (LM)	trainlm2	0.99775	0.95739	0.89542	0.95895

**Table 2:** Optimized GA process parameters and outputs corresponding to best fitted ANN training algorithm.

Sr. No.	ANN Training Algorithm	Optimized GA Value					Pullulan Yield (g/L) (Best Value)	Pullulan Yield (g/L) (Mean Value)
		Substrate ((g/L)	Time (h)	Temperature (°C)	pH	Agitation Speed (rpm)		
1	trainlm1	48.9	172.62	26.89	6.56	224.85	39.4918	39.3691
2	trainlm2	44.9	172.33	27.85	6.99	210.56	35.5672	35.5552

**Table 3:** Various chosen ANFIS training membership functions (MFs) and resultant optimized GA process parameters (epoch error, best value, and mean value for pullulan yield) and their respective yields.

Sr. No.	MF Type		Epoch 3: Error	Method Adopted to Generate FIS	Train FIS Optimized Method	Optimized GA Value					Pullulan Yield (g/L) (Best Value)	Pullulan Yield (g/L) (Mean Value)
						Substrate (g/L)	Time (h)	Temp. (◦C)	pH	Agitation Speed (rpm)		
1	Constant	gaussmf	6.1055 × 10^−5^	Grid-Partition	Hybrid	49.94	182.39	27.41	6.99	190.08	36.0788	36.0425
2	Constant	trimf	4.7389 × 10^−5^	Grid-Partition	Hybrid	49.7	180.04	27.45	6.99	190.29	35.8462	35.1299
3	Constant	gbellmf	0.00011749	Grid-Partition	Hybrid	49.94	181.51	27.39	6.98	190.07	35.8332	35.6952
4	Constant	gauss2mf	0.0002537	Grid-Partition	Hybrid	49.902	179.99	27.46	6.95	190.01	35.7434	35.7379
5	Constant	dsigmf	0.00032176	Grid-Partition	Hybrid	49.92	179.42	27.52	6.98	190.17	35.6809	35.6671

**Table 4:** Evaluation of optimization tools GA–ANN and GA–ANFIS with experimental data, with their optimized process parameters and outputs. Percentage accuracy of the 3 methods was also evaluated.

Sr. No	Optimization Tool/Method	Optimized Process Parameters					Predicted Pullulan Yield (g/L)	Experimental Pullulan Yield (g/L)	Percentage of Accuracy
		Substrate (g/L)	Time (h)	Temp (°C)	pH	Agitation Speed (rpm)			
1	Laboratory Fermentation	45.00	180	27.5	7.5	200	-	35.55	-
2	GA–ANN	48.90	172.62	26.89	6.56	224.85	39.4918	37.642 ± 0.521	95.32
3	GA–ANFIS	49.94	182.39	27.41	6.99	190.08	36.0788	35.652 ± 0.348	98.82
